# Effect of BL23 Acupressure on Pain Among Primiparous Women During the First Stage of Labor: A Randomized, Sham-Controlled Trial

**DOI:** 10.1155/prm/4486038

**Published:** 2025-08-20

**Authors:** Fahimeh Pishva, Reza Heshmat, Sedigheh Sedigh Mobarakabadi

**Affiliations:** ^1^Department of Midwifery and Reproductive Health, School of Nursing and Midwifery, Student Research Committee, Shahid Beheshti University of Medical Sciences, Tehran, Iran; ^2^Acupuncture, International School of Lyon, Sainte-Foy-lès-Lyon, France; ^3^Department of Midwifery and Reproductive Health, Midwifery and Reproductive Health Research Center, School of Nursing and Midwifery, Shahid Beheshti University of Medical Sciences, Tehran, Iran

**Keywords:** acupressure, BL23 point, childbirth, labor pain

## Abstract

**Introduction:** Labor pain is a well-known physiological phenomenon considered to be the most severe pain experienced by women of childbearing age. One nonpharmacological method used to alleviate labor pain is acupressure. This study aimed to examine the impact of acupressure on the BL23 points on pain intensity during the active phase of the first stage of labor.

**Method:** This randomized, sham-controlled clinical trial took place at Tehran Baharlu Hospital in Iran from August 23 to October 21, 2023. Ninety first-time pregnant women in active labor were randomly assigned to one of three groups: acupressure on BL23 (*n* = 30), sham acupressure (between the seventh and eighth thoracic vertebra, 2 Cun from the midline) (*n* = 30), and a control group (*n* = 30). The sham and acupressure groups received 60 min of acupressure at three different time points during cervical dilatation at 4–5 cm, 6–7 cm, and 8–10 cm. The control group received standard labor care. Pain severity was assessed using a Numerical Rating Scale before, 10 min after, and 20 min after the intervention at each time point.

**Result:** Pain intensity was significantly lower in the BL23 acupressure group compared to the control and sham groups at all three time points (*p* < 0.0001). Pain intensity decreased in the BL23 acupressure group after 10 min of intervention at all time points (*p* < 0.001) and continued to decrease throughout the intervention (*p* < 0.001). The reduction in pain in the BL23 acupressure group was evident before the start of the second and third interventions (*p*=0.33 *and* *p*=0.36, respectively). Twenty minutes of pressure on BL23 points at different dilatation stages were equally effective in reducing pain (*p*=0.13). No adverse effects on maternal and neonatal outcomes were observed in the BL23 acupressure group compared to the other groups (*p* > 0.05).

**Conclusion:** This study demonstrates that applying acupressure to the BL23 points during the active phase of the first stage of labor significantly reduces labor pain. However, the pain relief provided by this intervention is temporary, with its effects diminishing over time rather than offering permanent relief.

**Trial Registration:** Iranian Registry of Clinical Trials (IRCT): IRCT20230513058166N1

## 1. Introduction

Childbirth is a pivotal event in women's lives, frequently accompanied by intense pain that prompts many to seek effective pain relief [1, 2]. Both pharmacological and nonpharmacological approaches are available for managing labor pain. Increasingly, nonpharmacological methods are favored for their potential to reduce the medicalization of childbirth and promote a more natural birth experience [3].

Acupressure is a nonpharmacological technique that alleviates various types of pain by stimulating specific points on the body (acupoints) with targeted pressure. This method aims to restore the body's energy balance by activating the meridians' energy channels connected to particular organs [4, 5]. The BL23, one of the 12 Back Shu points, is recognized for its sedative properties. These points are located 1.5 Cun, approximately the combined width of the second and third fingers, laterally to the posterior median line [6].

BL23 points have been used in the treatment of pain syndromes such as low back and knee pain, genital pain, and postpartum perineal pain [7]. Additionally, BL23 is considered one of the 12 Back Shu points, known for its sedative properties [8]. During the active phase of the first stage of labor, women typically experience pain in the lower abdomen, iliac crests, and lower back, with pain sometimes radiating to the thighs and perineum toward the end of this phase [9].

Stux et al. (2003) suggest that stimulating proximal points (near the pain center) can trigger soothing effects through three centers: the spinal cord, midbrain, and hypothalamus–pituitary axis, leading to effective analgesic effects [10].

Acupuncture at BL23 points, when combined with other acupoints, serves as a valuable addition to managing labor pain and reducing the need for pharmaceutical or invasive pain relief methods [11]. The injection of a fentanyl–droperidol solution at BL23 points during the active labor phase has been linked to decreased pain intensity [12].

Although BL23 points have been used along with other acupoints to alleviate childbirth pain, the effectiveness of these points in relieving pain during labor is not well known. Further evidence-based research is necessary to determine the efficacy of acupressure at BL23 points for pain relief during labor. This study aimed to investigate the effect of acupressure on BL23 points on pain intensity during the active phase of the first stage of labor.

## 2. Methods

### 2.1. Setting and Participants

This study was a randomized, sham-controlled clinical trial conducted from August 23 to October 21, 2023, at Baharlu Hospital in Tehran, Iran. The inclusion criteria were as follows: participants had to be between 19 and 35 years; literate; experiencing their first birth with no history of more than one abortion; having a confirmed full-term pregnancy (verified by first-trimester ultrasound or the first day of the last menstrual period); carrying a singleton fetus in vertex presentation; having three or more contractions in 10 minutes; showing 4-5-cm cervical dilation; showing no indication of cephalopelvic disproportion; not having received any analgesic medication (chemical or herbal) in the last 8 h; not having any chronic illnesses such as cardiovascular disease, kidney disease, rheumatism, and epilepsy; not having any mental illness; not having experienced the loss of a spouse or close relative during pregnancy; not having any pregnancy complications such as preeclampsia or chorioamnionitis; and not having any obstetric complications at the time of study entry such as placental abruption or abnormal fetal heart rate. Additionally, women should not have any skin diseases or injuries near BL23 points. Exclusion criteria included the refusal of the mother to participate further in the study. Pain data from women with conditions such as cesarean birth, birth complications, and the use of pain medication before completing the three stages of the intervention were not statistically analyzed but were recorded and analyzed as secondary outcomes.

This study has been approved by the ethics committee of Shahid Beheshti University of Medical Sciences (IR.SBMU.PHARMACY.REC1402.047). The purpose of the study, details of the intervention, and the confidentiality of information were explained to all women. They were assured that they could withdraw from the study at any time during the intervention. The intervention researcher was blinded to the intervention group and study outcome at the start of the study. The women were randomly assigned to three groups, and informed consent was obtained from all participants. The research setting remained unchanged, except for the acupressure intervention for pain reduction. All participating mothers received the necessary routine care or treatment.

### 2.2. Sample Size and Randomization

Primiparous females who presented to the childbirth department to give birth were enrolled in the study. The initial sample size was calculated to be 27 participants per group, with a 5% Type I error rate (*α* = 0.05) and a 90% power (1-β = 0.90) [13]. This number was later adjusted to 30 participants per group to account for a potential 10% loss of samples. With three groups in the study (*r* = 3), an analysis of variance table was used to determine the appropriate sample size. To utilize this table, one must know the number of groups (*r* = 3) and the value of Δ/*σ*. Here, σ represents the standard deviation, and Δ is the difference between the highest and lowest mean pain quality scores, as identified by Ozgoli et al. [14]. The sampling method was initially chosen based on the research purpose, and individuals were then randomly assigned to groups using random allocation software [15]. Before the intervention, the third author used software to randomly sequence the order of individuals in the groups from 1 to 90. This order was then placed in an envelope for allocation concealment purposes. The researcher implementing the intervention (the first author) remained unaware of the group type of the research units until after assigning the women to the groups. Additionally, the participants receiving the sham and acupressure treatments were kept unaware of their assigned group by being blinded to the assignment until the end of the study.

### 2.3. Intervention

Before the study, the second author showed the first author how to locate the BL23 and sham acupoints and provide acupressure. Intrarater reliability was assessed between the two authors. After performing acupressure for 10 min on the right BL23 point of the subjects, the DeQi sensation set (including feelings of heat, cold, tingling, and heaviness) was checked off on a prepared checklist. Twenty minutes later, the first author provided 10 min of acupressure on the left BL23 point of the subjects. The DeQi sensation set was checked again on the checklist. The Kappa agreement coefficient and the McNemar test were used to examine the agreement between the two individuals. The Kappa coefficient was 1, and the McNemar test result with a *p* > 0.05 indicated high agreement between the acupuncturist and the first researcher.

Before starting the intervention, uterine contractions were assessed, and women were instructed on using the Numerical Rating Scale (NRS) with a sample provided for practice. Written consent was obtained, and women were randomly assigned to three groups: the BL23 acupressure group (aligned with the second and third lumbar vertebra, 1.5 Cun from the midline of the spine), the sham group (aligned 2 Cun from the midline of the vertebral column between the seventh and eighth thoracic vertebra), and the control group. Acupressure was administered at BL23 points for cervical dilations of 4-5 cm, 6-7 cm, and 8–10 cm for 20 min each, totaling 60 min. In the BL23 acupressure group, women reclined in a position allowing the researcher to access their backs. The researcher stood behind the mothers' heads and applied pressure with her thumbs on the women's BL23 points during contractions, pressing firmly and circularly until a color change occurred in the nail. This process was repeated at dilations of 6-7 and 8–10 cm. Acupressure on the sham group was carried out similarly. For the control group, the researcher stayed with the women who had reached 4-cm cervical dilation, providing emotional support at specific intervals for the same duration until the birth was complete.

### 2.4. Outcome Measurement

Pain intensity was measured as the primary outcome using a NRS ranging from 0 (*no pain*) to 10 (*worst imaginable pain*). Measurements were taken at three stages of cervical dilatation: 4-5 cm, 6-7 cm, and 8–10 cm, before and 10 and 20 min following the intervention in the acupressure groups. Pain intensity in these intervals was also assessed using an NRS in the control group.

Secondary outcomes included the type of birth (vaginal, cesarean, and instrumental), labor complications (fetal distress, placental issues, and prolonged labor), demand for pain relief medication, Apgar scores at 1 and 5 min, length of the second and third stages of labor, satisfaction with treatment (yes/to some extent/no; measurement: 12 h after birth), and pain relief effectiveness perceived by the participant (rated from 0 to 5; measurement: 12 h after birth). These outcomes were assessed using a checklist in three groups of women. Mothers were monitored for a period of up to 12 h after birth.

### 2.5. Data Analysis

The data were analyzed using SPSS Version 22. Normal distribution was checked using the Kolmogorov–Smirnov test. Group differences in the change in pain were compared by Friedman's test, and paired differences within groups were compared by Wilcoxon's test. The Kruskal–Wallis test was used to compare the effectiveness of pain relief between groups, as the data were not normally distributed. The two groups were compared using the Mann–Whitney test.

## 3. Result

### 3.1. Demographic and Basic Information

**Figure 1:**
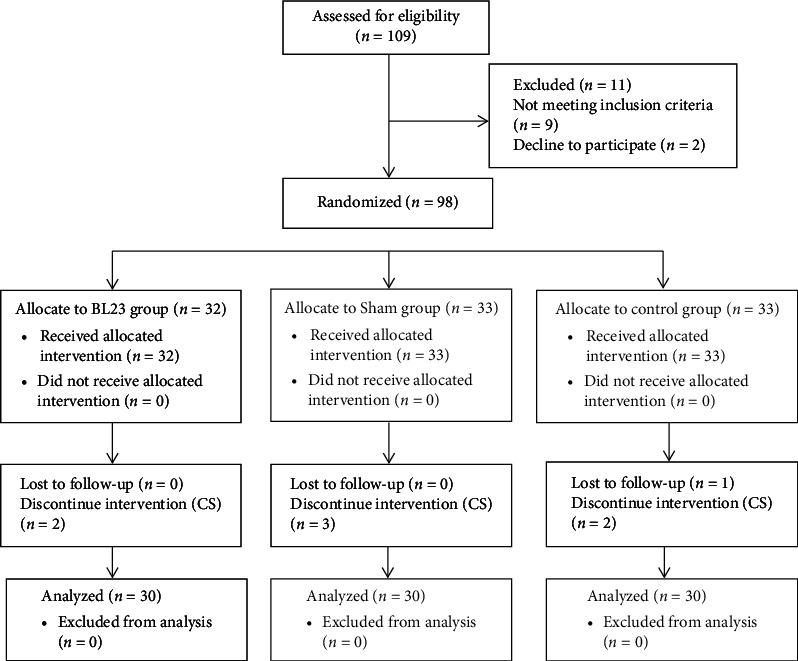
Participants' flow diagram.

Out of the 109 women evaluated, 98 were randomly assigned to three study groups. One mother from the control group dropped out, and 7 did not complete the study due to a cesarean birth. Data from 90 mothers were analyzed (Figure 1). The mean age of participating mothers was 28.10 ± 5.63. All of the mothers were married and living with their husbands. There were no demographic differences among the three groups (Table 1). All women received antenatal care. There was no statistically significant difference in the position of women during the interventions among the groups in the first, second, and third interventions (*p*=0.45,  *p*=0.29,  *and* *p*=0.79, respectively). Many women complained of pain focusing on the lower back or lower abdomen (*p*=0.77).  Table 2 shows the baseline characteristics of the three groups.

### 3.2. Pain Outcomes

The Kruskal–Wallis test revealed a significant difference in pain intensity among the three groups at 10 and 20 min after the intervention across all three time points at 4-5, 6-7, and 8–10 cm of cervical dilatations (*p* < 0.0001). A two-by-two analysis of the groups, using the Mann–Whitney test with Bonferroni correction, shows that the relief of pain intensity in the BL23 acupressure group was significantly greater compared to the sham and control groups (*p* < 0.001). During the first, second, and third intervention time points, there was no significant difference between the control and sham groups (*p*=0.86,  *p*=0.57,  *and* *p*=0.78, respectively). Based on the Friedman test, in the acupressure group, a significant difference was observed in BL23 points before, as well as 10 and 20 min after the intervention, with dilatations of 4-5, 6-7, and 8–10 cm (*p* < 0.001). The Wilcoxon test with Bonferroni correction showed a significant difference in all three time points of the intervention before and 10 min after (*p* < 0.001), before and 20 min after (*p* < 0.001), and 10 and 20 min after (*p*=0.0001,  *p*=0.002,  *and* *p*=0.0001 for the first, second, and third interventions, respectively). The acupressure group showed a higher average pain reduction during the third intervention, but statistical analysis did not find a significant difference in pain reduction across all three time points of the intervention (*p*=0.13) (Table 3).

### 3.3. Other Outcomes

One woman in the sham group and two women in the control and acupressure groups (a total of 5 women) underwent cesarean delivery. The reasons for this were fetal distress and prolonged labor. There were no significant differences in the duration of the second and third stages of labor (*p*=0.59 *and* *p*=0.34, respectively) or Apgar scores at 1 and 5 min between the groups (*p*=0.59 *and* *p*=0.34, respectively). One mother from the BL23 group, three mothers from the sham group, and five mothers from the control group requested medical interventions to alleviate labor pain. In the BL23 and control groups, one neonate required resuscitation. Women in the BL23 acupressure group perceived less pain than the other two groups (*p*=0.001). The level of satisfaction with the method was also higher in the BL23 group than in the other two groups (*p*=0.001). Birth outcomes did not differ significantly among the groups (Table 4).

## 4. Discussion

This study examined the effects of acupressure on BL23 points on pain intensity during the active phase of the first stage of labor. The results showed significant pain relief after each intervention period in the BL23 acupressure group compared to the sham and control groups. Acupressure on BL23 points was found to be effective in reducing pain intensity, with relief achieved after 10 min of continuous pressure and increasing with continued acupressure. The intervention was equally effective in reducing pain intensity during dilatations of 4-5, 6-7, and 8–10 cm.

Nesheim et al. used BL23 acupuncture and other points to reduce labor pain and decrease the need for meperidine [11]. Gohar and Taman found that acupressure on BL23 points reduces perineal pain postpartum [16]. Ghaemmaghami et al. showed that acupressure at BL23 reduces lower back pain after childbirth [17]. The lower back is a common location of pain during the active phase of labor, and toward the end of this phase, perineal pain is also characteristic [9]. Stimulating acupoints near the pain site activates endorphin-producing cells, inhibiting pain transmission in the spinal cord [10]. Considering this, it can be inferred that the findings of these studies might elucidate the outcomes of our research on pain relief.

The results of the study showed that after 10 min from the start of the intervention, there was a significant reduction in pain in the acupressure group at BL23 points. With the continuation of acupressure, a greater reduction in pain intensity was reported at the end of 20 min. Similar results were reported by Ozgoli et al. through the stimulation of BL32 acupoints, and by Azadeh et al. through the stimulation of the EX-B8 acupoint [18, 19]. More studies are needed to determine whether more than 20 minutes of acupressure can achieve a greater reduction in labor pain intensity.

Pain intensity was similar in all three groups before applying acupressure at 6-7 and 8–10 cm dilatations, indicating that the pain reduction from the interventions was not sustained. The type of pain changes as labor progresses, and the pain score also increases. Therefore, adapting to pain becomes increasingly difficult [9]. The effectiveness of acupressure decreases as pain becomes more intense and continuous. A study by Mady et al. found that pain intensity increased 60 min after applying acupressure on LI4 compared to before the intervention [20].

Chen et al. found from a meta-analysis of 13 clinical trials that acupressure provided significant and lasting pain relief for up to 60 min compared to control groups [21]. One possible reason for the similar pain intensity levels between the BL23 acupressure group and the sham and control groups before the second and third interventions could be the concurrent administration of oxytocin to most participants. However, more studies are needed to better understand the relationship between oxytocin administration and acupressure effects.

In our study, the mean pain intensity during the three stages of dilatation was −4.66 ± 1.02, −4.96 ± 0.85, and −5.40 ± 1.75. Ozgoli et al. also used the NRS tool to measure pain in three stages of acupressure intervention, reporting mean decreases in pain intensity of −3.06 ± 1.25, −3.46 ± 1.37, and −3.31 ± 1.69 in the LI4 group, and −3.69 ± 1.15, −4.34 ± 1.60, and −3.83 ± 1.75 in the BL32 group [14]. Raana and Fan conducted a meta-analysis showing a cumulative MD of acupressure pain reduction at Li4 and SP6 points with confidence intervals of −2.73 and −1.08 in the active phase and −5.03 and −1.02 in the transitional phase [22]. Our study demonstrated a significant reduction in pain intensity, suggesting the potential analgesic effect of these acupressure points during labor. Further research is needed to validate these findings and address potential limitations in blinding pain intensity measurements in our study.

In this study, we included a sham group to account for the placebo effect of acupressure. Though the decrease in pain level in the sham group was less than in the BL23 acupressure group, it was significant. Torkiyan et al. found similar results with a sham group [23]. We can infer that the pain relief associated with BL23 points is not solely due to psychological factors.

To address potential bias from the researcher's presence, a control group was included in the study. There was no significant difference in pain intensity. Previous research also used a control group to minimize bias, showing varying results at different stages of labor [14]. While some pain reduction was observed in our study, it was not statistically significant. Additional interventions may be necessary as labor progresses and pain intensifies.

During our study, acupressure on BL23 points did not alter the Apgar score of the infant, the second and third stage duration of labor, or delivery mode. Chen et al. reported a reduction in the duration of the second stage of labor in their meta-analysis [21].

The acupressure group reported greater satisfaction and experienced greater pain relief from the mother's perspective. Maddy et al. also found that mothers experienced high satisfaction and reduced pain intensity with LI4 point acupressure application [20].

The present study is one of the few studies that have focused on the effect of acupressure on BL23 points on labor pain intensity. One of the strengths of this study was that it had a control group and a sham group. The study was also conducted at three time points at different dilatations. Thus, the effectiveness of these points on pain intensity at different dilatations was determined. Examination of pain intensity before each stage of the intervention showed that acupressure does not have a consistent effect on labor pain intensity, especially when oxytocin is used in the labor process. One of the limitations of our study was the excessive use of oxytocin for labor augmentation. A second study is needed to establish the confounding influence of oxytocin on the analgesic effects of acupressure. Although the position of the fetal head in the mother's pelvis can affect the intensity of labor pain, we did not consider this item in our study. It is suggested that positions such as occiput posterior is separately examined in future studies.

## 5. Conclusion

The results of the research showed that acupressure at BL23 points during the active phase of the first stage of labor relieve pain but not enduringly. Whether the lack of persistence of the pain-relieving effect of acupressure at BL23 points is due to the nature of the acupressure at these points during labor or is due to the intensification of pain as a result of augmentation of labor with oxytocin requires further investigation.

## Figures and Tables

**Table 1:** Demographics characteristics.

Characteristics/groups	BL23 (*n* = 30)	Sham (*n* = 30)	Control (*n* = 30)	*p*
Age (years) (mean ± SD)	28.36 ± 5.49	27.93 ± 6.30	28.00 ± 5.21	0.19^a^
Women's education *n* (%)				
• Elementary	10 (33.34)	13 (43.34)	19 (63.34)	0.15^b^
• Secondary and high school	17 (56.66)	16 (53.33)	10 (33.33)	
• University	3 (10.00)	1 (3.33)	1 (3.33)	
Women's occupation *n* (%)				
• Housewife	28 (93.34)	28 (93.34)	28 (93.34)	0.80^b^
• Employed	2 (6.66)	2 (6.66)	2 (6.66)	
Housing *n* (%)				
• Rental	25 (83.34)	25 (83.34)	25 (83.34)	1.00^b^
• Private	5 (16.66)	5 (16.66)	5 (16.66)	
Income satisfaction *n* (%)				
• Less than enough	8 (26.66)	13 (43.33)	10 (33.33)	2.31^b^
• Enough	18 (60.00)	15 (50.00)	16 (53.33)	
• More than enough	4 (13.34)	2 (6.64)	4 (13.34)	
Nationality *n* (%)				
• Iranian	22 (73.33)	25 (83.33)	20 (66.66)	0.33^b^
• Afghanistanian	8 (26.66)	5 (16.66)	10 (33.33)	

^a^Kruskal–Wallis test.

^b^chi square.

**Table 2:** Baseline characteristics.

Characteristics/groups	BL23 (*n* = 30)	Sham (*n* = 30)	Control (*n* = 30)	*p*
Gestational age (weeks) (mean ± SD)	39.28 ± 0.75	39.34 ± 0.81	39.37 ± 0.81	0.89^a^
Number of pregnancies *n* (%)				
• 1	29 (96.66)	30 (100)	29 (96.66)	—
• 2	1 (3.34)	0 (0.00)	1 (3.34)	
Satisfaction with pregnancy *n* (%)				
• Yes	26 (86.66)	29 (96.66)	26 (86.66)	0.32^b^
• No	4 (13.34)	1 (3.34)	4 (13.34)	
Number of prenatal care (mean ± SD)	7.03 ± 0.76	7.03 ± 0.88	6.90 ± 1.29	0.94^a^
Childbirth preparation class *n* (%)				
• Yes	5 (16.66)	5 (16.66)	1 (3.34)	0.19^b^
• No	25 (83.33)	25 (83.33)	29 (96.66)	
Bishop score (mean ± SD)	3.80 ± 1.74	3.13 ± 1.54	3.23 ± 1.43	0.28^a^
Pattern of uterus contractions (mean ± SD)				
• Frequency	3.90 ± 4.92	3.03 ± 0.18	3.03 ± 0.18	1.00^a^
• Duration (second)	31.16 ± 6.11	31.53 ± 7.18	34.00 ± 8.24	0.44^a^
Contraction intensity *n* (%)				
• Moderate	24 (80.00)	24 (80.00)	23 (76.66)	0.93^a^
• Severe	6 (20.00)	6 (20.00)	7 (23.34)	
Amniotic membrane *n* (%)				
• Intact	12 (40.00)	13 (43.33)	14 (46.66)	0.87^a^
• Ruptured	18 (60.00)	17 (56.67)	16 (53.34)	
Fetal heart rate (mean ± SD)	141.26 ± 7.53	141.03 ± 9.24	140.93 ± 7.40	0.89^a^
Use of oxytocin *n* (%)	28 (93.33)	30 (100.00)	28 (93.33)	0.35^b^
Mean use of oxytocin (minute) (mean ± SD) (%)	411.83 ± 169.81	480.83 ± 124.10	446.00 ± 167.34	0.32^a^
Mean amount of oxytocin (mean ± SD) (Unit)	3.87 ± 2.12	4.50 ± 1.52	4.00 ± 2.03	0.40^c^
Vital signs (mean ± SD)				
• Blood pressure (systole)	113.40 ± 6.49	116.20 ± 4.76	115.86 ± 5.17	0.12^a^
• Blood pressure (diastole)	72.86 ± 6.02	75.73 ± 4.97	76.10 ± 4.99	0.06^a^
• Temperature	36.85 ± 0.33	36.82 ± 0.33	36.71 ± 0.29	0.18^a^
• Respiratory	17.50 ± 1.22	17.23 ± 1.04	17.70 ± 1.20	0.34^a^
• Pulse rate	82.33 ± 5.33	81.56 ± 4.32	83.13 ± 4.04	0.37^a^

^a^KruskaltWallis test.

^b^chi square.

^c^ANOVA.

**Table 3:** Comparison of the mean pain scores before and after each intervention within and among the groups.

Stages groups	4-5 cm				*p* ^a^	6-7 cm				*p* ^a^	8–10 cm				*p* ^a^
	Before	After 10 min	After 20 min	Change before after 20 min		Before	After 10 min	After 20 min	Change before and after 20 min		Before	After 10 min	After 20 min	Change before and after 20 min	
BL23 (*n* = 30)	9.63 ± 0.71	5.53 ± 1.00	4.96 ± 0.76	−4.66 ± 1.02	0.001	10.00 ± 0.0	5.36 ± 0.71	5.03 ± 0.85	−4.96 ±0 .85	0.001	9.66 ± 0.60	5.40 ± 6.21	4.90 ± 0.66	−5.40 ± 1.75	0.001
Sham (*n* = 30)	9.36 ± 0.66	9.10 ± 0.84	9.06 ± 0.82	−0.30 ± 0.74	0.46	9.83 ± 0.15	9.63 ± 0.55	9.66 ± 0.47	−0.26 ± 0.52	0.004	9.65 ± 0.71	9.73 ± 0.44	9.70 ± 0.46	−0.30 ± 0.46	0.001
Control (*n* = 30)	9.30 ± 0.65	9.20 ± 0.66	9.13 ± 0.68	−0.16 ± 0.64	0.250	9.93 ± 0.25	9.86 ± 0.43	9.73 ± 0.52	−0.20 ± 0.61	0.097	9.73 ± 0.58	9.86 ± 0.35	9.80 ± 0.40	−0.20 ± 0.40	0.009
*p* ^b^	0.06	0.001	0.001	0.0001		0.33	0.001	0.001	0.0001		0.36	0.001	0.001	0.0001	

^a^Friedman's test.

^b^Kruskal–Wallis test.

**Table 4:** Comparison of secondary outcome variables and satisfaction among the groups.

Outcomes/groups	BL23 (*n* = 30)	Sham (*n* = 30)	Control (*n* = 30)	*p*
The duration of the second stage of labor (min) (mean ± SD)	42.23 ± 8.08	43.36 ± 18.75	43.53 ± 8.52	0.59^a^
The duration of the third stage of labor (min) (mean ± SD)	5.83 ± 2.33	5.56 ± 2.76	5.76 ± 1.88	0.34^a^

**Outcomes/groups**	**BL23 (*n* = 32)**	**Sham (*n* = 33)**	**Control (*n* = 33)**	**p**

Birth weight (gram) (mean ± SD)	3268.00 ± 284.35	3196.96 ± 365.44	3129.0 ± 304.77	0.23^a^
Apgar score at the first min (mean ± SD)	9.03 ± 0.18	9.06 ± 0.25	9.10 ± 0.30	0.58^a^
Apgar score at the fifth min (mean ± SD)	10.0 ± 0.00	10.0 ± 0.00	10.0 ± 0.00	
Level of pain relief perceived by the women (mean ± SD)	5.00 ± 0.00	2.23 ± 1.00	2.53 ± 1.00	0.001^a^
Satisfaction (%)				
• Yes	29 (96.66)	6 (20.00)	5 (16.66)	0.001^b^
• To some extent	1 (3.33)	15 (50.00)	7 (23.33)	
• No	0 (0.00)	9 (30.00)	18 (60.00)	

^a^Kruskal–Wallis test.

^b^chi square.

## Data Availability

The data that support the findings of this study are available on request from the corresponding author.

## Nomenclature

BL23: Bladder 23
RCT: Randomized controlled trial
NRS: Numerical Rating Scale

## References

[1] Pietrzak J., Mędrzycka-Dąbrowska W., Tomaszek L., Grzybowska M. E. (2022). A Cross-Sectional Survey of Labor Pain Control and Women’s Satisfaction. *International Journal of Environmental Research and Public Health*.

[2] Rachmawati I. N. (2012). Maternal Reflection on Labour Pain Management and Influencing Factors. *British Journal of Midwifery*.

[3] Sedigh M. S., Mirzaiinajmabadi K., Ghazi T. M., Esmaily H. (2017). Predictors of Mode of Childbirth Based on Medicalized Maternal Care: A Cross-Sectional Study.

[4] Young A., Shipe M., Smith M. (2021). Non-Pharmacological Pain Managment in Labor: a Systematic Review.

[5] Klein B. E., Gouveia H. G. (2022). Use of Non-pharmacological Pain Relief Methods in Labor. *Cogitare Enfermagem*.

[6] Organization W. H. (2008). *WHO Standard Acupuncture Point Locations in the Western Pacific Region*.

[7] Akbarzade M., Ghaemmaghami M., Yazdanpanahi Z., Zare N., Mohagheghzadeh A., Azizi A. (2016). Comparison of the Effect of Dry Cupping Therapy and Acupressure at BL23 Point on Intensity of Postpartum Perineal Pain Based on the Short Form of Mcgill Pain Questionnaire. *Journal of Reproduction and Infertility*.

[8] Associated (Back-Shu) Points The Chiropractic Resource Organization. https://chiro.org/acupuncture/ABSTRACTS/FILES/Alarm_Points.pdf.

[9] Lowdermilk D. L., Cashion K., Perry S. E., Alden K. R., Olshansky E. (2019). *Maternity and Women’s Health Care E-Book*.

[10] Stux G., Berman B., Pomeranz B. (2003). *Basics of Acupuncture*.

[11] Nesheim B.-I., Kinge R., Berg B. (2003). Acupuncture During Labor Can Reduce the Use of Meperidine: a Controlled Clinical Study. *The Clinical Journal of Pain*.

[12] Zhu H.-X., Yao Y., Wu Y.-S., Liu Y., Yan L.-R., Su X.-J. (2013). Influence of Acupoint Injection with Small Dose of fentanyl-droperidol Mixed Liquor on Labor Analgesia and Level of Stress Hormone in Parturient. *Zhongguo Zhen jiu= Chinese Acupuncture & Moxibustion*.

[13] Cohen J. (2013). *Statistical Power Analysis for the Behavioral Sciences: Routledge*.

[14] Ozgoli G., Sedigh Mobarakabadi S., Heshmat R., Alavi Majd H., Sheikhan Z. (2016). Effect of LI4 and BL32 Acupressure on Labor Pain and Delivery Outcome in the First Stage of Labor in Primiparous Women: a Randomized Controlled Trial. *Complementary Therapies in Medicine*.

[15] Saghaei M. (2004). Random Allocation Software for Parallel Group Randomized Trials. *BMC Medical Research Methodology*.

[16] Gohar I. E., Taman A. H. S. (2022). Effect of Acupressure Applied to Bl23 Point Versus Crushed Ice Application on Postpartum Perineal Pain Intensity. *Assiut Scientific Nursing Journal*.

[17] Ghaemmaghami M., Akbarzadeh M., Yazdanpanahi Z., Zara N., Azizi A., Mohagheghzadeh A. (2014). Comparison of Dry Cupping Therapy and BL 23 Acupressure Point on the Severity of Lower Back Pain After Delivery in Nulliparous Women Based on the Visual Assessment Scale in 2012. *SSU_Journals*.

[18] Ozgoli G., Sedigh S., Heshmat R., Alavi M. H. (2010). Effect of Right Hand Hegu Acupressure on Pain Intensity of Active Phase of Labor in Primiparous Women.

[19] Azadeh H., Heshmat R., Nasiri M., Azarkish F., Sedigh Mobarakabadi S. (2025). The Effect of EX‐B8 Acupressure on Labor Pain: a Randomized, Single‐Blind, Sham‐Controlled Trial. *Pain Research and Management*.

[20] Mady M. M., Aly I. K., Abu-Elmagd E. H., Aly S. G. (2024). Effect of Acupressure on Labor Pain for Women During First Stage of Normal Labor. *Journal of Medicine in Scientific Research*.

[21] Chen Y., Xiang X.-Y., Chin K. H. R. (2021). Acupressure for Labor Pain Management: a Systematic Review and meta-analysis of Randomized Controlled Trials. *Acupuncture in Medicine*.

[22] Raana H. N., Fan X.-N. (2020). The Effect of Acupressure on Pain Reduction During First Stage of Labour: a Systematic Review and meta-analysis. *Complementary Therapies in Clinical Practice*.

[23] Torkiyan H., Sedigh Mobarakabadi S., Heshmat R., Khajavi A., Ozgoli G. (2021). The Effect of GB21 Acupressure on Pain Intensity in the First Stage of Labor in Primiparous Women: a Randomized Controlled Trial. *Complementary Therapies in Medicine*.

